# Future of Endemic Flora of Biodiversity Hotspots in India

**DOI:** 10.1371/journal.pone.0115264

**Published:** 2014-12-12

**Authors:** Vishwas Sudhir Chitale, Mukund Dev Behera, Partha Sarthi Roy

**Affiliations:** 1 Centre for Oceans, Rivers, Atmosphere and Land Sciences, Indian Institute of Technology Kharagpur, West Bengal, India; 2 Geospatial Solutions, International Centre for Integrated Mountain Development (ICIMOD), Kathmandu, Nepal; 3 University Center for Earth and Space Sciences, University of Hyderabad, Hyderabad, Andhra Pradesh, India; Fondazione Edmund Mach, Research and Innovation Centre, Italy

## Abstract

India is one of the 12 mega biodiversity countries of the world, which represents 11% of world's flora in about 2.4% of global land mass. Approximately 28% of the total Indian flora and 33% of angiosperms occurring in India are endemic. Higher human population density in biodiversity hotspots in India puts undue pressure on these sensitive eco-regions. In the present study, we predict the future distribution of 637 endemic plant species from three biodiversity hotspots in India; Himalaya, Western Ghats, Indo-Burma, based on A1B scenario for year 2050 and 2080. We develop individual variable based models as well as mixed models in MaxEnt by combining ten least co-related bioclimatic variables, two disturbance variables and one physiography variable as predictor variables. The projected changes suggest that the endemic flora will be adversely impacted, even under such a moderate climate scenario. The future distribution is predicted to shift in northern and north-eastern direction in Himalaya and Indo-Burma, while in southern and south-western direction in Western Ghats, due to cooler climatic conditions in these regions. In the future distribution of endemic plants, we observe a significant shift and reduction in the distribution range compared to the present distribution. The model predicts a 23.99% range reduction and a 7.70% range expansion in future distribution by 2050, while a 41.34% range reduction and a 24.10% range expansion by 2080. Integration of disturbance and physiography variables along with bioclimatic variables in the models improved the prediction accuracy. Mixed models provide most accurate results for most of the combinations of climatic and non-climatic variables as compared to individual variable based models. We conclude that a) regions with cooler climates and higher moisture availability could serve as refugia for endemic plants in future climatic conditions; b) mixed models provide more accurate results, compared to single variable based models.

## Introduction

### Global climate change and vegetation shift

Global change, including the changes in atmospheric composition, climate and land use, has modified and affected the climate system as well natural and anthropogenic-influenced ecosystems [1]. Anthropogenic climate change is a major component of global change, which includes various issues such as the intensity and frequency of extreme events, the magnitude and rate of change, the change of mean climate state and climate variability, long-term and short-term changes, and rapid or abrupt changes [2]. These changes will ultimately affect the ecosystems of the world, primarily influencing the distribution and growth of plants. Elevated CO_2_ and NO_2_ have been reported to affect the distribution of plants by controlling the plant growth [3]. Global temperatures are expected to rise by up to 4°C by 2100 [4]. However the increase is neither temporally nor spatially uniform, where areas with above average warming co-occur with areas with minor warming or even slight cooling. Globally, climate change over last few decades has caused numerous shifts in the distribution of plant species [5]–[8]. Tropical ecosystems are already experiencing higher degree of species extinction as well as alterations in species distribution due to rising temperatures, erratic rainfall, altered climatic extremes, where plants have been observed to be more sensitive to the variations in the seasonal extremes; other factors also involve anthropogenic disturbances such as conversion of land cover and forest fragmentation [9]–[14]. Projecting future changes in the distribution of endemic flora is a crucial step towards planning and mitigating the impacts of climate change on biodiversity [15].

### Species distribution modeling

Modeling species' ecological niche and their potential distribution proves to be a powerful tool for ecologists and conservation biologists to assess the impacts of climate change. Predictive models of species geographic distributions play a crucial role in a variety of applications in ecology and conservation [16]. Attempts to estimate the future effects of global climate change on biodiversity based on environmental envelope models have often been reported [17]–[18]. Presence-only modeling methods are advantageous over presence/absence modeling methods, because the data requirement of predictor variables is less. A variety of statistical methods are available to construct species distribution models [19]–[20]. MaxEnt is a bioclimatic model, which deals with *presence only* data and has a number of aspects that makes it well-suited for species distribution modeling [21]–[22]. For better assessment of climate change impacts, it is necessary to go beyond the naive assumption that plant distributions are mechanistically controlled by simple climate variables, and recognize the role of complex non-climatic factors [23]. Various researchers have attempted to include non-climatic variables such as land cover [24]–[25], altitude [26]–[27], slope, fragmentation [28], human population [29]. Integration of non-climatic variables along with dynamic climatic variables could provide better insight into the climate change impacts on endemic plants.

### Endemic plant species

The plant species, which are unique to a defined geographic unit such as an island/nation or habitat type and are not found elsewhere, are known as endemic plant species. Physical, climatic, and biological factors can contribute to endemism of plants. Species with narrow distribution range and/or fewer individuals are considered to be the most prone to extinction due to changing climatic conditions and competition by alien species. Endemic species have long been targets for conservation efforts, because they are not found anywhere else in the world and if lost from their native habitat they will be lost forever. Myers et al., [30] hypothesized that conservation of endemic species could result in conservation of species rich landscapes. Assessing present and future distribution of endemic species would be crucial contribution for their conservation planning and management.

### Biodiversity hotspots in India

India possesses abundant biodiversity owing to its larger climatic and topographic gradient. Indian forests cover 22.5% of country's geographical area and harbor more than 17000 angiosperms [31]–[32]. In 2006, *Conservation International* demarcated 34 global ‘Biodiversity Hotspots’, four of which partly fall within Indian political boundaries: 1) Himalaya, 2) Western Ghats, 3) Indo-Burma and 4) Sundaland [30]. Roy et al., [13] observed that highly fragmented forests across the Indian landscape harbor a number of endemic species, which need to be conserved. Higher rates of human population growth in India put these ecoregions at a risk of extinction due to over-increasing human interference, fragmentation, deforestation, and expansion of agricultural lands in the forested landscapes [33]. Although, these hotspots cover significant portion of global biodiversity hotspots, they have been insufficiently studied and less understood [34].

## Methods

### Study area

**Figure 1:**
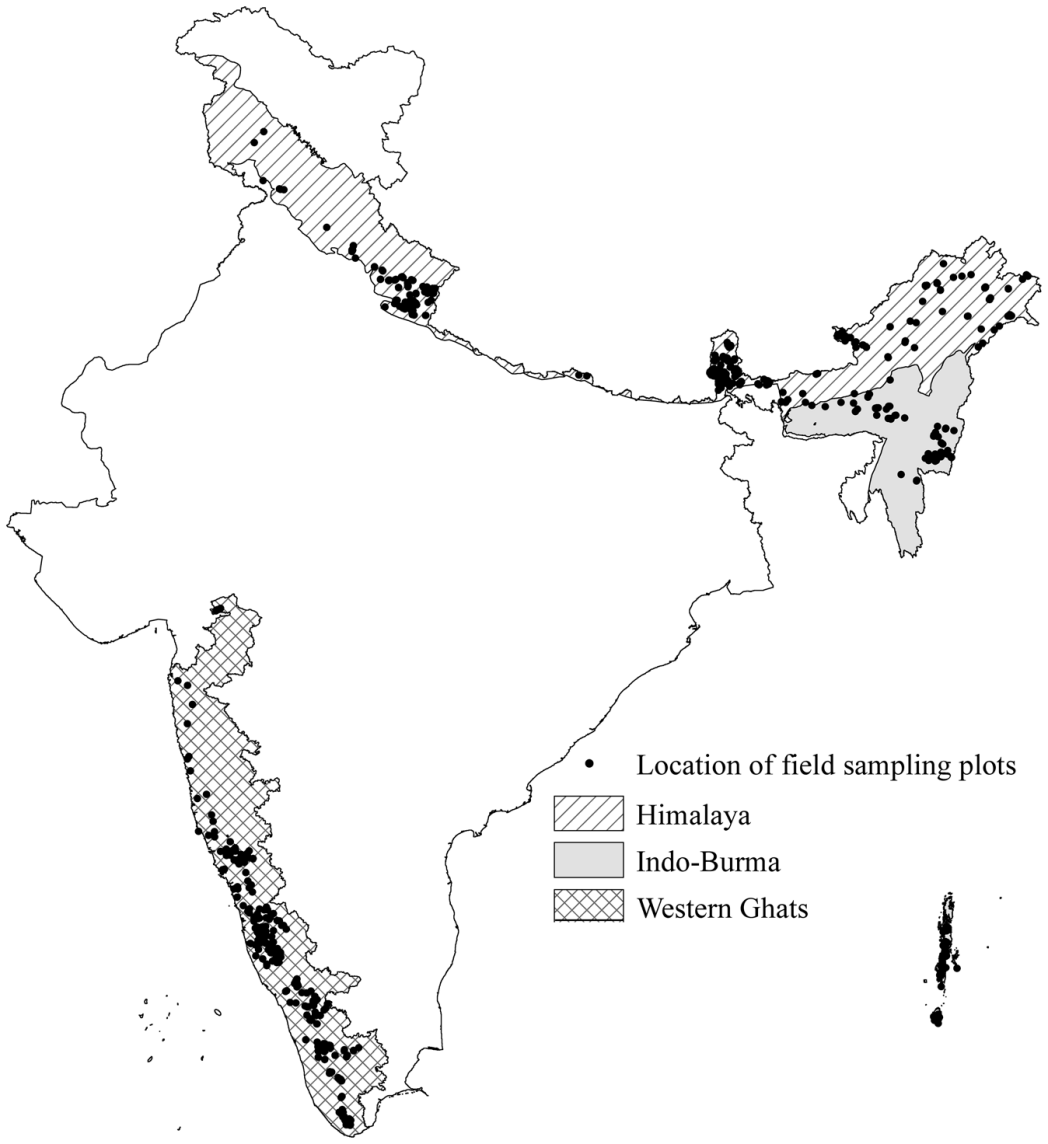
Location of field sampling plots in biodiversity hotspots in India a) Himalaya, b) Western Ghats, c) Indo-Burma.

Three biodiversity hotspots considered in the present study; Himalaya, Western Ghats and Indo-Burma are located in three different eco-regions of India and experience distinct climatic patterns (Fig. 1). Himalaya, covering *c*. 329,109.22 km^2^ (44.37% of global biodiversity hotspot), is located between 25°39′28″ and 35°49′48″ N Latitude; 73°08′04″ and 97°24′44″ E Longitude, along the northern boundary of India. The climate is sub-alpine in western Himalaya, while it is sub-tropical to temperate in eastern Himalaya, where annual temperature and precipitation fluctuate from 5 °C and 1200 mm in western Himalaya and 10 °C and 3500 mm in eastern Himalaya respectively. The elevational gradient ranging from 500–8800 m results in a diversity of ecosystems that range in only a couple of hundred kilometers; from alluvial grasslands and subtropical broadleaf forests along the foothills to temperate broadleaf forests in the mid hills, mixed conifer and conifer forests in the higher hills, and alpine meadows above the tree line [35].

The Western Ghats covering *c*. 125,035.19 km^2^ (64.95% area of global biodiversity hotspot), is located between 8°04′45″ and 22°01′40″ N Latitude; 72°38′34″ and 78°28′18″ E Longitude, along an elevational gradient of 300–2700 m. In general, the mean temperature of the coldest month ranges from 25°C at sea level to 11 °C at 2400 m. The western slopes of the mountains in Western Ghats experience heavy annual rainfall, while the eastern slopes are drier; rainfall also decreases from south to north. The wide variation of rainfall patterns in the Western Ghats, coupled with the region's complex geography, produces a great variety of vegetation types [36]. These include scrub forests in the low-lying rain-shadow areas and the plains, deciduous and tropical rainforests up to about 1,500 meters, and a unique mosaic of montane forests and rolling grasslands above 1,500 meters. Faced with tremendous pressure from anthropogenic factors, the forests of the Western Ghats have been dramatically impacted by the demands for agricultural land, timber and built-up land.

Indo-Burma, covering *c*. 121,721.19 km^2^ (5.13% area of global biodiversity hotspot), is located between 10°30′34″-26°55′50″ N Latitude; 89°51′16″-95°22′48″ E Longitude, along an elevational gradient of 750–2300 m. A wide diversity of ecosystems is represented in this hotspot, ranging from mixed wet evergreen, dry evergreen, deciduous, and montane forests, which have resulted from the complex topographic and climatic gradients. Indo-Burma is one of the most threatened biodiversity hotspots in the world, due to higher rates of resource exploitation and habitat loss.

### Data and predictor variables

The vegetation data used in our analysis originates from stratified random sampling based nested quadrates of 0.04 ha laid during 1998 to 2008, as a part of the ‘*biodiversity characterisation at landscape level project*’ [37] conducted for whole India. We utilize the geographic locations of 324 endemic plants species from Himalaya, 217 from Western Ghats and 96 from Indo-Burma collected under 1264, 1004 and 1114 plots respectively (Fig. 1, S1 Table). Only species with more than 15 occurrences are considered during the study.

We initially select 35 bioclimatic (C) variables from CliMond database [38] that have been demonstrated to have a direct ecophysiological impact on plant species (S2 Table). To avoid the bias introduced due to multi-colinearity of climate variables, we remove 25 highly correlated climate variables (with spearman's correlation coefficient >0.70); retaining least correlated 10 climate variables (S2 Table). Altitude, as a function of physiography (P), is derived from Shuttle Radar Topographic Mission (SRTM) digital elevation model (DEM) [39] in a GIS environment ArcGIS 9.2. These variables have already been used successfully in several modeling studies conducted across the globe (e.g., [40], [7], [41]).

Two disturbance (D) variables of year 2010: a) forest fragmentation, and b) human population density, are utilized in the study. Forest fragmentation reduces the size of local habitat patches and the connectivity among patches, thus increasing edge and isolation effects, which in turn make species more susceptible to extinction due to environmental variability, demographic stochasticity and genetic influences [28]. Vegetation type map of India of year 2008, generated during ‘*biodiversity characterisation at landscape level project*’ [37] is updated for year 2010, using multitemporal satellite data of Landsat Thematic Mapper by following the same classification scheme and methodology (For detailed methodology please refer Ref. [37]). Forest fragmentation was calculated at 500 m spatial resolution based on vegetation type map of year 2010 using Spatial Analysis and Modelling package (S1, [13]) and finally a predictor layer is generated in the GIS environment ArcGIS 9.2 [42].

Human population in these hotspots pose risk to the biodiversity of these eco-regions by over-logging, burning, grazing, mining and commercial hunting that have extracted or degraded natural resources, abetted biological invasion or polluted soil and water resources [36]. For generating the raster layer of human population of 2010 based on the Census of India data, we followed the methodology proposed by SEDAC for generating Gridded Human Population of the World (GPW). The gridded human population layers generated by SEDAC till year 2000 are based on actual human population obtained through surveys, while layers for time periods recent than year 2000 are based on the estimates, which could have introduced errors in the analysis of the present study. Therefore, we did not use the estimated human population provided by SEDAC for year 2010, instead we used the survey based human population data obtained from Census of India report 2011 and methodology provided by SEDAC to create district wise rasterized human population of year 2010. Spatial grids of 5 km^2^ resolution were created covering the total geographical area of the districts falling under the hotspots. The total human population under the districts was equally divided under the number of grids falling in each district, which when summed for a district results in total population of the corresponding district [33], [43]. Disturbance and physiographic predictor variables are resampled at 10′ spatial resolution in ArcGIS 9.2 using majority resampling method to match with the spatial extent of climatic variables acquired from CliMond database.

### Modelling approach

We create models using MaxEnt software version 3.3.3e downloaded from Princeton University website (http://www.cs.princeton.edu/~schapire/maxent/, [21]) to assess the contribution of climatic, physiographic and disturbance factors in the future distribution of endemic plants. The models are created with the following combinations: a) climate variables only (C), b) climate + disturbance (C+D), c) climate + physiography (C+P), d) climate + disturbance + physiography (C+D+P) for year 2050 and 2080; e) physiography only (P), f) disturbance only (D), and g) physiography + disturbance (P+D) are created for the present time period and utilized for both future predictions. The AUC value of P, D, and P+D models remains same for both time periods; whereas the AUC of mixed models of P and D with climatic variables varies for both prediction years.

Prediction accuracy and significance of the models is assessed based on three commonly used measures: a) Area Under Curve (AUC)), b) sensitivity (percentage of correctly classified presences) and c) specificity (percentage of correctly classified absences). These measures are estimated from 3250 random splits of the original dataset into a calibration subset with 70% of the data and a validation subset with 30% of the data, which is used by the model to assess the statistical significance. We also followed the jackknife (also called ‘leave-one-out’) procedure and the results were obtained in the form of area under curve (AUC) indicating the significance of the environmental variables together and also as an individual variable. Best suitable model for each hotspot is identified based on the model accuracy.

## Results and Discussion

### Prediction accuracy

**Figure 2:**
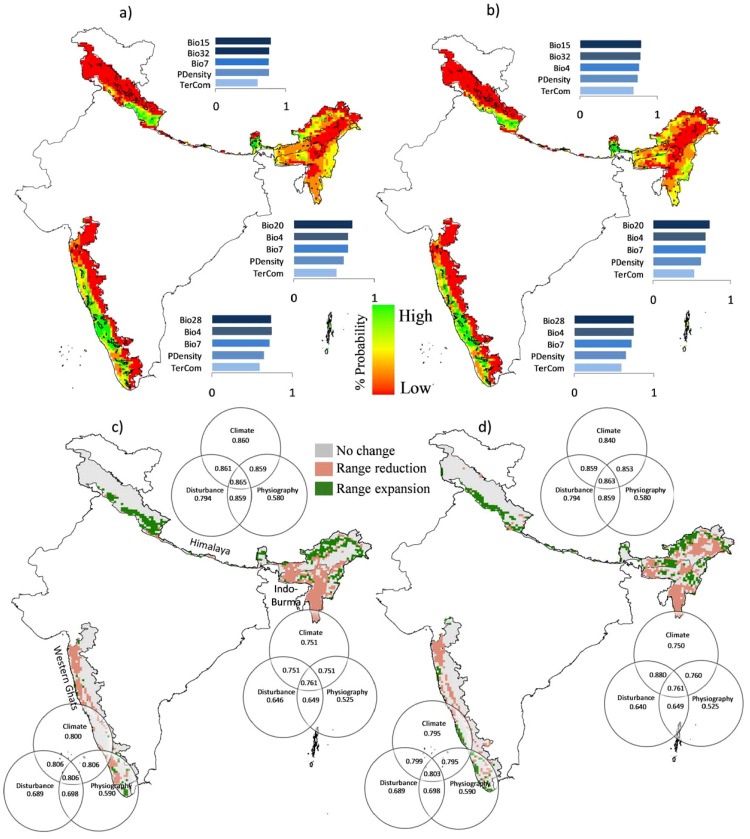
Future distribution of endemic plants in Indian biodiversity hotspots under A1B scenario with contribution of predictor variables, a) for year 2050, b) 2080; change in distribution range during c) year 2050, d) 2080. Venn diagrams indicate the model performance of individual variable based models and mixed models for individual hotspot (Bio4: Temperature seasonality, Bio7: Temperature annual range, Bio15: Precipitation seasonality, Bio20: Annual mean radiation, Bio28: Annual mean moisture index, Bio32: Mean moisture index of wettest quarter, PDensity: Human population density, TerCom: Terrain complexity).

Prediction accuracy for the C+D+P model for all hotspots is observed to be the highest for both 2050 and 2080 time periods, compared to individual variable based C, D and P models (Fig. 2). We find the AUC values for C+D+P model for Himalaya to be 0.865 and 0.863, for Western Ghats 0.806 and 0.803, and for Indo-Burma 0.761 and 0.761 for years 2050 and 2080, respectively. Highest AUC in C model is observed for Himalaya (0.860 and 0.840), followed by Western Ghats (0.800 and 0.795) and Indo-Burma (0.751 and 0.750) for years 2050 and 2080, respectively (Fig. 2 c and d). In D model, highest AUC is also observed for Himalaya (0.794), followed by Western Ghats (0.689) and Indo-Burma (0.646), while highest AUC in P model is observed for Western Ghats (0.590), followed by Himalaya (0.580) and Indo-Burma (0.525). Individual variable based disturbance and physiography models are observed to be less significant as compared to the climate models. However, addition of disturbance and physiography variables to the climate models improved the prediction accuracy compared to the individual variable based models. This could be due to the fact that the three categories of variables selected in the study are the most relevant variables in predicting the future distribution of endemic plants in tropical countries. Integrating fragmentation and human population density as predictor variables provide a better insight in the way these factors might affect the future distribution of endemic plants. It is a well-known fact that other factors, in addition to climate, affect the species distribution, such as competition [44] and soil type [45]. Given all these non-climatic determinants of species distribution, it is not surprising that only a relatively lesser variation in species distribution is explained by the models utilized in the present study. Future work may involve prediction of future distribution of the endemic flora by integrating some more non-climatic variables and by considering dispersal mechanisms of plants. Some progress is being made in adding non-climatic variables in SDMs for improving prediction accuracy. The model accuracy could also be improved by utilizing fine scale bioclimatic variables e.g., Worldclim [46], however the variables such as moisture index and solar radiation are not available in these datasets.

### Future distribution of endemic plants

We utilize SDMs to explore the influence of climate change and anthropogenic disturbance on future species distributions. The projected changes suggest that the endemic flora will be adversely impacted, even under such a moderate climate scenario. The overall future distribution is predicted to shift in the northern and north-eastern direction, compared to the present distribution (Fig. 2). In western Himalaya, the species are predicted to move in the eastern direction, with most of high ranked cells falling in the Uttarakhand state. The regions in western Himalaya, where the species are predicted to move in future climatic conditions are already experiencing highest anomaly in mean annual temperature (0.90–1.35°C.) over the last century, compared to any other parts of Himalaya. This could pose additional stress of unfavorable climatic conditions in these regions. If the shift of these endemic plants of Himalaya continues in the northern and north-eastern direction, the Indian portion of Himalaya may lose a few species due to species’ migration to Nepal, Bhutan and China. Apart from climate change, excessive human interference and pollution due to tourism is another major problem in this region. The number of tourists every year should be restricted, in order to reduce the stress on the endemic flora, which is sensitive to even a slight change in the climatic conditions.

The range reduction during the period from 2050–2080 is predicted to be greater than for the period 2010–2050, which could be due to the fact that very high rise in temperature is anticipated in 2080. In eastern Himalaya, the species are predicted to accumulate in the states of Sikkim and Arunachal Pradesh, as they have the coldest climatic conditions in eastern Himalaya. The forests of these regions already accommodate higher number of endemic species, which may undergo undue stress due to the invasion of ‘moving’ species. The eastward shift in the future distribution could also lead to break-up of present day flora of eastern Himalaya. The future distribution in eastern Himalaya for year 2080, particularly in Sikkim, is predicted to shift in the northern direction, compared to that during 2050.

In the Western Ghats, the species are predicted to shift in the southern and south-western direction towards the coastal regions (Fig. 2). This could be attributed to the fact that coastal regions are expected to experience less warming than in-land regions. The regions situated in southern and south-eastern parts of Western Ghats experience higher annual rainfall and low mean monthly temperature, compared to other parts in the hotspot. These regions could serve as ‘refugia’ in the future climatic conditions. Our results are in line with those obtained by Das et al., [47], where they observed a declining trend in SR from south to north and west to east direction in Western Ghats. Apart from topographic variability, the reasons for higher temperature in northern parts of Western Ghats could be: a) higher human population density in metropolitan cities like Mumbai resulting in higher atmospheric pollution, b) most of the forests along the northern parts of Western Ghats are highly fragmented, which also causes rise in temperature. Western Ghats hotspot accommodates the higher human population density than any other hotspot in the world and with only 9% of the forests under protected area network the endemic flora of Western Ghats is under the threat of extinction. In Indo-Burma, the future distribution is predicted to shift in the eastern and south-eastern direction (Fig. 2). As per the future distribution for year 2050, the endemic flora is predicted to accumulate in central parts of Indo-Burma, while an eastward shift is predicted in the distribution for 2080. The model predicts a larger amount of range reduction than range expansion in the future distribution in Indo-Burma. The range reduction in areas such as Island archipelago of Andaman could threaten the endemic flora, resulting in their extinction due to lack of favorable areas for migration. India's forests are under tremendous pressure due to higher human population density within and around the forested landscapes causing excessive human interference and habitat fragmentation. The three hotspots considered in the study face different challenges to cope up with biodiversity degradation and loss. Alpine grasslands and scrublands in the western Himalaya harbor a higher degree of endemic plants compared to forests. However, these grasslands are being exploited to cater the needs such as food and fodder for livestock. In eastern Himalaya, the biodiversity is under threat due to illegal hunting [48] of animals to cater higher demands of animal fur, skin and bones from across the International boundary. The forests of Western Ghats face stress due to higher human population as well as mining activities [47]. The forests of Indo-Burma are fragmented due to conventional agricultural practices such as shifting cultivation/slash and burn [49].

### Hotspot-wise variation in predictor variables

We observe different patterns in contribution of climate variables in the future distribution of endemic species across the three hotspots (Fig. 2a and b). Annual mean moisture index and temperature seasonality are the most significant climate variables in Western Ghats, precipitation seasonality and mean moisture index of wettest quarter are significant in Himalaya, and annual mean radiation and temperature seasonality are significant in Indo-Burma. Apart from these variables, temperature annual range is observed to be a significant variable in the future prediction in all three hotspots for the years 2050 and 2080. This highlights the physiognomic variations in the three hotspots, which are located in different bio-geographic zones.

Western Ghats exhibits future distribution of species towards the areas with lower temperature seasonality and higher annual mean moisture index. The reason behind significant contribution of lower temperature seasonality is because regions such as Western Ghats, which are located along coastlines, are expected to experience less warming compared to inland areas in the future. The movement of endemic plants towards the southern Western Ghats, which experience greater annual mean moisture index, highlights its contribution in impacting their future distribution. Himalaya on the other hand, exhibits cooler and drier climate, than the other two hotspots, where the role of moisture is highlighted through precipitation seasonality and mean moisture index of wettest quarter. Moderate mean annual radiation and moderate temperature range are expected to support species distribution in Indo Burma in future climate. Various researchers have explained the role of temperature and precipitation in assessing the future distribution of species. However, our results indicate that annual mean radiation and annual mean moisture might also play a significant role in deriving the patterns of species distribution.

### Range dynamics and refugia

Our results support the fact that cooler climates and moisture rich areas could serve as refugia for biodiversity. Western Ghats hotspot accommodates highest plant endemism compared to other two hotspots, which supports the fact that areas with lower temperature seasonality promote multidimensionality and complexity of biotic interactions, all of which supports high biological diversity. Higher temperature may be associated with greater complexity in possible ways of life, thus enhancing diversity. It is expected that climate change in the regions with warmer climates could increase the length of landscape-scale temperature gradient, which accommodates higher environmental diversity and supports higher landscape-scale species diversity. We observe varied patterns in future distribution for the years 2050 and 2080, which highlight the transition zones along the south-western parts of Western Ghats, indicating range expansion, and south-eastern parts, indicating range reduction. However, the distribution along southern parts of western Himalaya indicate range expansion, while southern parts of eastern Himalaya (prominently in Arunachal Pradesh) indicated range reduction (Fig. 2c and d). In Indo-Burma, very few patches are found to be suitable for range expansion under future distribution, while range reduction is observed in most of parts of the hotspot. We predict a reduction in distribution range by 23.99% and 41.34%, and an expansion by 7.70% and 24.10% in prediction of 2050 and 2080 respectively.

Bioclimatic envelope models have been widely implemented to assess possible impacts of climate change on distribution of species [20]. Present study focuses on projecting the future distribution of endemic plants with respect to climatic, anthropogenic disturbance, and physiographic variables. Although, it is a well-known fact that plant species respond to climate change, it is a difficult task to assess the way in which they are responding to it. Also the dispersal capacities of plants differ from each other, which result in differential migration lag. Nonetheless, the study has limits in considering plant dispersal mechanisms, soil nutrient limitations, and competition, which falls beyond the scope of the present study and could not be addressed in a combined model approach. However, addressing these questions could be attempted in the future studies. Nonetheless, to answer these questions we need to run the individual species specific models, which might provide species specific responses to climate change. However, combined modeling approach could prove to be advantageous while predicting future distribution at broader scales (e.g., hotspot level). It is more useful than species specific models in region specific conservation prioritization, which has been recommended as better conservation strategy than species specific conservation [23].

Integration of non-climatic variables along with climate variables improve the predictive power of the models. Fragmentation of forested landscapes could lead to mass scale extinction of endemic flora of India's biodiversity hotspots. Temperatures are expected to rise by 1.4–6.4°C during the 21st century [50], which will place additional stress on species that prefer cooler temperatures and are currently restricted to interglacial refugia. Identifying refugia from climate change is an important step in conservation planning, which provide suitable habitats for climate change threatened species [51], [52]. As far as we are aware, this is the first-ever published study of the impacts of climate change and anthropogenic disturbance on the endemic flora of biodiversity hotspots in India. The magnitude of our range shifts is in line with the studies conducted across the globe. Kueppers et al., [5] projected a northward shift in two Californian Oak species, while Lenihan et al., [53] predicted a northward shift in the future distribution of broadleaved forests. We predict a 23.99% and a 41.34% range reduction during 2050 and 2080 respectively. Most of the areas with predicted range reduction in the future distribution fall in high altitude ecosystems, which is similar to those reported by Loarie et al., [7] where they project >80% range reduction in 66% of California's endemic flora. There is an urgent need to expand the existing PA network in order to cope up with the ‘movement of species’. Similar studies could be conducted for assessing the impacts of climate change on mammals, birds and amphibians, which also form major components of these biodiversity hotspots.

## Conclusions

The results of our study have implications for conservation of endemic flora of biodiversity hotspots, given the urgency with which we must identify areas that need to be protected. Based on the present study we conclude that: a) regions with cooler climates and higher moisture availability could serve as refugia for endemic species under future climatic conditions, b) mixed models provide better insight into the impacts of climate change on endemic plants, as compared to single variable based models. Prediction accuracy of the species distribution models depend on the factors like spatial resolution, size of the study area, method of choice, and quality of input datasets [38], [46], [54], [55]. The predictions based on the SDMs play a crucial role in conservation and planning, considering the projected impacts of climate change on the endemic flora. Similar models for other taxonomic groups would be useful for the conservation of whole biogeographic region. We suggest following conservation implications to address climate change induced alterations in the species distribution: a) assisted migration to support better survival of species into suitable habitats, b) expansion of protected area network in the areas of future distribution, and c) promote landscape connectivity.

## Supporting Information

S1 Table List of endemic species considered in the study.
(DOC)

S2 Table List of predictor variables.
(DOC)

S1 File Forest fragmentation.
(DOCX)
