# Supplementary material for: Future of Endemic Flora of Biodiversity Hotspots in India
Source: PLoS One. 2014 Dec 12;9(12):e115264. doi: 10.1371/journal.pone.0115264 (PMC4264876; doi:10.1371/journal.pone.0115264)
Supplement: S1 Table — List of endemic species considered in the study. (DOC) [file pone.0115264.s001.doc]

| Table S1 List of endemic plants considered in the study | | | |
| --- | --- | --- | --- |
| Endemic plant species of Himalaya | | | |
| **Sr. No.** | **Species Name** | **Family** | **Habit** |
| 1 | *Abies densa* | Pinaceae | T |
| 2 | *Acanthus leucostachyus* | Acanthaceae | T |
| 3 | *Acer caesium* | Aceraceae | T |
| 4 | *Acer campbellii* | Aceraceae | T |
| 5 | *Acer oblongum* | Aceraceae | T |
| 6 | *Achyranthes aspera* | Amaranthaceae | H |
| 7 | *Aconitum assamicum* | Ranunculaceae | H |
| 8 | *Aconitum ferox* | Ranunculaceae | H |
| 9 | *Aconitum nagarum* | Ranunculaceae | H |
| 10 | *Actephila excelsa* | Phyllanthaceae | S |
| 11 | *Aesculus indica* | Sapindaceae | S |
| 12 | *Agapetes griffithii* | Vacciniaceae | S |
| 13 | *Agapetes incurvata* | Vacciniaceae | H |
| 14 | *Alseodaphne khasyana* | Lauraceae | T |
| 15 | *Alysicarpus vaginalis* | Fabaceae | H |
| 16 | *Amomum subulatum* | Zingiberaceae | T |
| 17 | *Ampelocissus latifolia* | Vitaceae | H |
| 18 | *Androsace lanuginosa* | Primulaceae | H |
| 19 | *Anemone elongata* | Ranunculaceae | T |
| 20 | *Anemone griffithii* | Ranunculaceae | H |
| 21 | *Antidesma bunius* | Euphorbiaceae | T |
| 22 | *Aquilaria malaccensis* | Thymelaeaceae | H |
| 23 | *Aquilegia pubiflora* | Ranunculaceae | S |
| 24 | *Archidendron bigeminum* | Fabaceae | T |
| 25 | *Ardisia griffithii* | Myrsinaceae | S |
| 26 | *Ardisia pedunculosa* | Myrsinaceae | T |
| 27 | *Areca nagensis* | Arecaceae | T |
| 28 | *Arenaria glanduligera* | Caryophyllaceae | H |
| 29 | *Arisaema intermedium* | Araceae | H |
| 30 | *Aristolochia cathcartii* | Aristolochiaceae | S |
| 31 | *Artemisia roxburghiana* | Asteraceae | H |
| 32 | *Arundinaria callosa* | Poaceae | S |
| 33 | *Arundinaria falcata* | Poaceae | S |
| 34 | *Arundinaria maling* | Poaceae | H |
| 35 | *Aster molliusculus* | Asteraceae | H |
| 36 | *Aster peduncularis* | Asteraceae | H |
| 37 | *Aster thomsonii* | Asteraceae | H |
| 38 | *Astragalus candolleanus* | Fabaceae | H |
| 39 | *Astragalus himalayanus* | Fabaceae | H |
| 40 | *Atalantia wightii* | Rutaceae | H |
| 41 | *Athyrium mackinnoniorum* | Athyriaceae | H |
| 42 | *Bambusa arundinacea* | Poaceae | S |
| 43 | *Bambusa balcooa* | Poaceae | S |
| 44 | *Bambusa tulda* | Poaceae | T |
| 45 | *Bauhinia semla* | Fabaceae | S |
| 46 | *Bauhinia vahlii* | Fabaceae | T |
| 47 | *Begonia rex* | Begoniaceae | H |
| 48 | *Begonia sikkimensis* | Begoniaceae | T |
| 49 | *Beilschmiedia brandisii* | Lauraceae | T |
| 50 | *Beilschmiedia pseudo-microcarpa* | Lauraceae | T |
| **Sr. No.** | **Species Name** | **Family** | **Habit** |
| 51 | *Benkara fasciculata* | Rubiaceae | H |
| 52 | *Berberis aristata* | Berberidaceae | S |
| 53 | *Berberis chitria* | Berberidaceae | S |
| 54 | *Berberis insignis* | Berberidaceae | S |
| 55 | *Berberis jaeschkeana* | Berberidaceae | S |
| 56 | *Berberis lycium* | Berberidaceae | S |
| 57 | *Berberis macrosepala* | Berberidaceae | S |
| 58 | *Bergenia ciliata* | Saxifragaceae | H |
| 59 | *Bergenia stracheyi* | Saxifragaceae | T |
| 60 | *Brassaiopsis mitis* | Araliaceae | T |
| 61 | *Bupleurum hamiltonii* | Apiaceae | H |
| 62 | *Butea monosperma* | Fabaceae | T |
| 63 | *Buxus wallichiana* | Buxaceae | S |
| 64 | *Calamus gracilis* | Arecaceae | T |
| 65 | *Callicarpa vestita* | Verbenaceae | S |
| 66 | *Capparis assamica* | Capparaceae | S |
| 67 | *Cardamine scoriarum* | Brassicaceae | H |
| 68 | *Carex obscura* | Cyperaceae | H |
| 69 | *Caryopteris odorata* | Verbenaceae | S |
| 70 | *Caryota obtusa* | Arecaceae | T |
| 71 | *Cautleya robusta* | Zingiberaceae | H |
| 72 | *Cautleya spicata* | Zingiberaceae | H |
| 73 | *Cedrus deodara* | Pinaceae | T |
| 74 | *Celtis australis* | Cannabaceae | T |
| 75 | *Cephalotaxus griffithii* | Cephalotaxaceae | T |
| 76 | *Chirita mishmiensis* | Gesneriaceae | H |
| 77 | *Chirita oblongifolia* | Gesneriaceae | H |
| 78 | *Cinnamomum tamala* | Lauraceae | T |
| 79 | *Cirsium verutum* | Asteraceae | H |
| 80 | *Claoxylon rostratum* | Euphorbiaceae | S |
| 81 | *Clematis acuminata* | Ranunculaceae | C |
| 82 | *Coelogyne fuscescens* | Orchidaceae | S |
| 83 | *Coffea benghalensis* | Rubiaceae | S |
| 84 | *Coffea khasiana* | Rubiaceae | S |
| 85 | *Colebrookea oppositifolia* | Lamiaceae | S |
| 86 | *Coptis teeta* | Ranunculaceae | T |
| 87 | *Corylus jacquemontii* | Corylaceae | T |
| 88 | *Crotalaria calycina* | Fabaceae | H |
| 89 | *Cryptocarya amygdalina* | Lauraceae | T |
| 90 | *Cupressus torulosa* | Cupressaceae | H |
| 91 | *Cyclostemon subsessilis* | Euphorbiaceae | H |
| 92 | *Cymbidium dayanum* | Orchidaceae | H |
| 93 | *Dalbergia sissoo* | Fabaceae | C |
| 94 | *Danthonia cachemyriana* | Poaceae | C |
| 95 | *Daphne papyracea* | Thymelaeaceae | T |
| 96 | *Delphinium denudatum* | Ranunculaceae | H |
| 97 | *Dendrobium spathaceum* | Orchidaceae | S |
| 98 | *Dendrocalamus hamiltonii* | Poaceae | S |
| 99 | *Dendrocnide meyeniana* | Urticaceae | S |
| 100 | *Desmos lawii* | Annonaceae | H |
|  |  |  |  |
|  |  |  |  |
|  |  |  |  |
| **Sr. No.** | **Species Name** | **Family** | **Habit** |
| 101 | *Deutzia staminea* | Hydrangeaceae | S |
| 102 | *Didymocarpus pulchera* | Gesneriaceae | H |
| 103 | *Dioscorea belophylla* | Dioscoreaceae | C |
| 104 | *Dioscorea bulbifera* | Dioscoreaceae | C |
| 105 | *Dioscorea deltoidea* | Dioscoreaceae | C |
| 106 | *Diospyros montana* | Ebenaceae | T |
| 107 | *Diplazium esculentum* | Athyriaceae | H |
| 108 | *Diploknema butyracea* | Sapotaceae | H |
| 109 | *Dracaena petiolata* | Asperagaceae | S |
| 110 | *Dysoxylum reticulatum* | Meliaceae | T |
| 111 | *Elaeocarpus glandulosus* | Elaeocarpaceae | T |
| 112 | *Elaeocarpus sikkimensis* | Elaeocarpaceae | T |
| 113 | *Elatostema decipiens* | Urticaceae | H |
| 114 | *Elatostema griffithii* | Urticaceae | H |
| 115 | *Elatostema lineolatum* | Urticaceae | H |
| 116 | *Elatostema papillosum* | Urticaceae | H |
| 117 | *Ellertonia rheedei* | Lamiaceae | H |
| 118 | *Enkianthus himalaicus* | Ericaceae | H |
| 119 | *Epilobium latifolium* | Onagraceae | H |
| 120 | *Eragrostis nutans* | Poaceae | H |
| 121 | *Eriobotrya anguistissima* | Rosaceae | S |
| 122 | *Eulalia mollis* | Poaceae | H |
| 123 | *Euonymus attenuatus* | Celastraceae | S |
| 124 | *Euonymus echinatus* | Celastraceae | S |
| 125 | *Euonymus indicus* | Celastraceae | T |
| 126 | *Euphorbia royleana* | Euphorbiaceae | H |
| 127 | *Evodia lunu-ankenda* | Rutaceae | T |
| 128 | *Flacourtia indica* | Flacourtiaceae | S |
| 129 | *Garuga pinnata* | Burseraceae | H |
| 130 | *Gaultheria fragrantissima* | Ericaceae | T |
| 131 | *Gaultheria nummularioides* | Ericaceae | T |
| 132 | *Gentiana speciosa* | Gentianaceae | H |
| 133 | *Geranium wallichianum* | Geraniaceae | H |
| 134 | *Geum elatum* | Rosaceae | S |
| 135 | *Globba multiflora* | Zingiberaceae | H |
| 136 | *Globba pauciflora* | Zingiberaceae | S |
| 137 | *Globba racemosa* | Zingiberaceae | T |
| 138 | *Glochidion calocarpum* | Euphorbiaceae | T |
| 139 | *Glochidion khasicum* | Euphorbiaceae | S |
| 140 | *Glochidion malabaricum* | Euphorbiaceae | S |
| 141 | *Grewia optiva* | Tiliaceae | S |
| 142 | *Hedychium spicatum* | Zingiberaceae | H |
| 143 | *Hedychium venustum* | Zingiberaceae | H |
| 144 | *Himalayacalamus falconeri* | Poaceae | S |
| 145 | *Hoya lobbii* | Asclepiadaceae | C |
| 146 | *Hypericum oblongifolium* | Hypericaceae | S |
| 147 | *Ilex dipyrena* | Aquifoliaceae | T |
| 148 | *Ilex embelioides* | Aquifoliaceae | T |
| 149 | *Ilex insignis* | Aquifoliaceae | T |
| 150 | *Ilex khasiana* | Aquifoliaceae | T |
|  |  |  |  |
|  |  |  |  |
| **Sr. No.** | ***Species Name*** | **Family** | **Habit** |
| 151 | *Ilex venulosa* | Aquifoliaceae | T |
| 152 | *Impatiens discolor* | Balsaminaceae | H |
| 153 | *Impatiens jurpia* | Balsaminaceae | H |
| 154 | *Impatiens minor* | Balsaminaceae | S |
| 155 | *Impatiens scabrida* | Balsaminaceae | H |
| 156 | *Impatiens stenantha* | Balsaminaceae | H |
| 157 | *Indoneesiella echioides* | Acanthaceae | T |
| 158 | *Ixora finlaysoniana* | Rubiaceae | S |
| 159 | *Ixora subsessilis* | Rubiaceae | T |
| 160 | *Jasminum multiflorum* | Oleaceae | S |
| 161 | *Jasminum scandens* | Oleaceae | H |
| 162 | *Justicia vasculosa* | Acanthaceae | H |
| 163 | *Khasiaclunea oligocephala* | Rubiaceae | S |
| 164 | *Knema cinerea* | Myristicaceae | T |
| 165 | *Lagerstroemia microcarpa* | Pinaceae | T |
| 166 | *Lasianthus biermanii* | Rubiaceae | S |
| 167 | *Lasianthus hookeri* | Rubiaceae | S |
| 168 | *Lasianthus tubiferus* | Rubiaceae | S |
| 169 | *Leptodermis lanceolata* | Rubiaceae | S |
| 170 | *Lindelofia longiflora* | Boraginaceae | H |
| 171 | *Lindera latifolia* | Lauraceae | S |
| 172 | *Litsea meissneri* | Lauraceae | C |
| 173 | *Livistona jenkinsiana* | Arecaceae | H |
| 174 | *Macaranga indica* | Euphorbiaceae | T |
| 175 | *Magnolia pterocarpa* | Magnoliaceae | S |
| 176 | *Malaxis muscifera* | Orchidaceae | H |
| 177 | *Mallotus tetracoccus* | Euphorbiaceae | T |
| 178 | *Maytenus heyneana* | Celastraceae | S |
| 179 | *Mesua ferrea* | Clusiaceae | T |
| 180 | *Michelia baillonii* | Magnoliaceae | T |
| 181 | *Miliusa macrocarpa* | Annonaceae | H |
| 182 | *Morina longifolia* | Morinaceae | T |
| 183 | *Morus serrata* | Moraceae | S |
| 184 | *Musa velutina* | Musaceae | S |
| 185 | *Nepeta hindostana* | Lamiaceae | H |
| 186 | *Nepeta leucophylla* | Lamiaceae | H |
| 187 | *Ochna obtusata* | Ochnaceae | S |
| 188 | *Olea glandulifera* | Oleaceae | S |
| 189 | *Ophiorrhiza fasciculata* | Rubiaceae | H |
| 190 | *Ophiorrhiza treutleri* | Rubiaceae | S |
| 191 | *Oxyspora vagans* | Melastomataceae | C |
| 192 | *Oxytropis cachemirica* | Fabaceae | H |
| 193 | *Pachylarnax pleiocarpa* | Magnoliaceae | S |
| 194 | *Parthenocissus semicordata* | Vitaceae | H |
| 195 | *Pennisetum flaccidum* | Poaceae | H |
| 196 | *Persea gamblei* | Lauraceae | T |
| 197 | *Persea gammieana* | Lauraceae | T |
| 198 | *Persea globularia* | Lauraceae | T |
| 199 | *Persicaria amplexicaulis* | Polygonaceae | H |
| 200 | *Peucedanum nagpurense* | Apiaceae | H |
|  |  |  |  |
|  |  |  |  |
| **Sr. No.** | **Species Name** | **Family** | **Habit** |
| 201 | *Phlogacanthus tubiflorus* | Lauraceae | T |
| 202 | *Phoebe cooperiana* | Lauraceae | T |
| 203 | *Pholidota articulata* | Orchidaceae | C |
| 204 | *Picea smithiana* | Pinaceae | T |
| 205 | *Pieris formosa* | Ericaceae | T |
| 206 | *Pilea insolens* | Urticaceae | H |
| 207 | *Pilea ternifolia* | Urticaceae | S |
| 208 | *Pinus roxburghii* | Pinaceae | T |
| 209 | *Pinus wallichiana* | Pinaceae | C |
| 210 | *Piper petiolatum* | Piperaceae | C |
| 211 | *Poeciloneuron indicum* | Clusiaceae | H |
| 212 | *Pogostemon benghalense* | Lamiaceae | S |
| 213 | *Polyalthia fragrans* | Annonaceae | T |
| 214 | *Polygala tricholopha* | Polygalaceae | H |
| 215 | *Polygonatum brevistylum* | Liliaceae | H |
| 216 | *Polygonum affine* | Polygonaceae | T |
| 217 | *Polygonum recumbens* | Polygonaceae | H |
| 218 | *Polygonum rude* | Polygonaceae | H |
| 219 | *Potentilla argyrophylla* | Rosaceae | H |
| 220 | *Potentilla atrisanguinea* | Rosaceae | H |
| 221 | *Potentilla microphylla* | Rosaceae | H |
| 222 | *Potentilla nepalensis* | Rosaceae | H |
| 223 | *Premna barbata* | Verbenaceae | T |
| 224 | *Premna bengalensis* | Verbenaceae | T |
| 225 | *Premna milleflora* | Verbenaceae | T |
| 226 | *Prunus arborea* | Rosaceae | T |
| 227 | *Prunus punctata* | Rosaceae | T |
| 228 | *Pseudophegopteris levingei* | Thelypteridaceae | H |
| 229 | *Psychotria adenophylla* | Rubiaceae | H |
| 230 | *Psychotria anamallayana* | Rubiaceae | S |
| 231 | *Psychotria monticola* | Rubiaceae | S |
| 232 | *Pyrus pashia* | Rosaceae | T |
| 233 | *Quercus ferox* | Fagaceae | T |
| 234 | *Quercus floribunda* | Fagaceae | T |
| 235 | *Reinwardtiodendron anamalaiense* | Meliaceae | T |
| 236 | *Rhamnus procumbens* | Rhamnaceae | S |
| 237 | *Rhamnus triqueter* | Rhamnaceae | T |
| 238 | *Rhaphidophora calophyllum* | Araceae | H |
| 239 | *Rhaphidophora glauca* | Araceae | H |
| 240 | *Rhaphidophora hookeri* | Araceae | H |
| 241 | *Rheum australe* | Polygonaceae | H |
| 242 | *Rheum spiciforme* | Polygonaceae | H |
| 243 | *Rheum webbianum* | Polygonaceae | H |
| 244 | *Rhododendron anthopogon* | Ericaceae | S |
| 245 | *Rhododendron arboreum* | Ericaceae | S |
| 246 | *Rhododendron barbatum* | Ericaceae | S |
| 247 | *Rhododendron campanulatum* | Ericaceae | T |
| 248 | *Rhododendron campylocarpum* | Ericaceae | T |
| 249 | *Rhododendron dalhousiae* | Ericaceae | S |
| 250 | *Rhododendron edgeworthii* | Ericaceae | S |
|  |  |  |  |
|  |  |  |  |
| **Sr. No.** | **Species Name** | **Family** | **Habit** |
| 251 | *Rhododendron grande* | Ericaceae | T |
| 252 | *Rhododendron griffithianum* | Ericaceae | T |
| 253 | *Rhododendron hodgsonii* | Ericaceae | S |
| 254 | *Rhododendron lepidotum* | Ericaceae | S |
| 255 | *Rhododendron megeratum* | Ericaceae | T |
| 256 | *Rhododendron wightii* | Ericaceae | S |
| 257 | *Rhus hookeri* | Anacardiaceae | T |
| 258 | *Rhynchotechum alternifolium* | Gesneriaceae | S |
| 259 | *Rhynchotechum vestitum* | Gesneriaceae | S |
| 260 | *Ribes griffithii* | Grossulariaceae | S |
| 261 | *Rosa macrophylla* | Rosaceae | S |
| 262 | *Rubia manjith* | Rubiaceae | H |
| 263 | *Rubus birmanicus* | Rosaceae | H |
| 264 | *Rubus hamiltonii* | Rosaceae | T |
| 265 | *Rubus lucens* | Rosaceae | S |
| 266 | *Rubus paniculatus* | Rosaceae | S |
| 267 | *Rubus rosaefolius* | Rosaceae | S |
| 268 | *Salix sikkimensis* | Salicaceae | T |
| 269 | *Salvia lanata* | Lamiaceae | T |
| 270 | *Sapium eugeniaefolium* | Euphorbiaceae | S |
| 271 | *Sarcosperma arboreum* | Sapotaceae | C |
| 272 | *Saurauia roxburghii* | Actinidiaceae | S |
| 273 | *Schima wallichii* | Theaceae | S |
| 274 | *Schisandra grandiflora* | Schisandraceae | H |
| 275 | *Scrophularia elatior* | Scrophulariaceae | H |
| 276 | *Scutellaria discolor* | Lamiaceae | S |
| 277 | *Scutellaria repens* | Lamiaceae | H |
| 278 | *Selaginella adunca* | Selaginellaceae | H |
| 279 | *Selinum vaginatum* | Apiaceae | H |
| 280 | *Selinum wallichianum* | Apiaceae | T |
| 281 | *Senecio graciliflorus* | Asteraceae | T |
| 282 | *Senecio pachycarpus* | Asteraceae | T |
| 283 | *Senecio quinquelobus* | Asteraceae | T |
| 284 | *Shorea assamica* | Dipterocarpaceae | T |
| 285 | *Shorea robusta* | Dipterocarpaceae | T |
| 286 | *Sloanea sterculiacea* | Elaeocarpaceae | H |
| 287 | *Smilax glaucophylla* | Smilacaceae | S |
| 288 | *Smilax rigida* | Smilacaceae | S |
| 289 | *Spondias pinnata* | Anacardiaceae | H |
| 290 | *Stellaria monosperma* | Caryophyllaceae | H |
| 291 | *Sterculia villosa* | Sterculiaceae | T |
| 292 | *Strobilanthes angustifrons* | Acanthaceae | H |
| 293 | *Strobilanthes atropurpureus* | Acanthaceae | H |
| 294 | *Strobilanthes dalhousianus* | Acanthaceae | H |
| 295 | *Strobilanthes discolor* | Acanthaceae | S |
| 296 | *Strobilanthes extensus* | Acanthaceae | H |
| 297 | *Strobilanthes secunda* | Acanthaceae | S |
| 298 | *Sumbaviopsis albicans* | Euphorbiaceae | H |
| 299 | *Swertia ciliata* | Gentianaceae | H |
| 300 | *Symplocos cochinchinensis* | Symplocaceae | T |
|  |  |  |  |
|  |  |  |  |
| **Sr. No.** | **Species Name** | **Family** | **Habit** |
| 301 | *Symplocos laurina* | Symplocaceae | S |
| 302 | *Syzygium balsameum* | Myrtaceae | T |
| 303 | *Syzygium kurzii* | Myrtaceae | T |
| 304 | *Tabernaemontana heyneana* | Apocynaceae | H |
| 305 | *Tetrastigma dubium* | Vitaceae | C |
| 306 | *Tetrastigma planicaule* | Vitaceae | C |
| 307 | *Thalictrum radiatum* | Ranunculaceae | H |
| 308 | *Themeda onathera* | Poaceae | H |
| 309 | *Trachelospermum lucidum* | Apocynaceae | H |
| 310 | *Trichodesma khasianum* | Boraginaceae | H |
| 311 | *Tsuga dumosa* | Pinaceae | T |
| 312 | *Tylophora himalaica* | Asclepiadaceae | C |
| 313 | *Ulmus wallichiana* | Ulmaceae | S |
| 314 | *Uvaria eucinata* | Annonaceae | S |
| 315 | *Vaccinium retusum* | Vacciniaceae | S |
| 316 | *Vaccinium vacciniaccum* | Vacciniaceae | S |
| 317 | *Vaccinium venosum* | Vacciniaceae | H |
| 318 | *Ventilago denticulata* | Rhamnaceae | C |
| 319 | *Ventilago maderaspatana* | Rhamnaceae | T |
| 320 | *Viburnum cotinifolium* | Caprifoliaceae | S |
| 321 | *Viola pilosa* | Violaceae | H |
| 322 | *Viola sikkimensis* | Violaceae | C |
| 323 | *Waldheimia tomentosa* | Asteraceae | S |
| 324 | *Wallichia densiflora* | Arecaceae | T |

|  | | | |
| --- | --- | --- | --- |
| **Endemic plant species of Western Ghats** | | | |
| **Sr. No.** | **Species Name** | **Family** | **Lifeform** |
| 1 | *Achyranthes aspera* | Amaranthaceae | H |
| 2 | *Actephila excelsa* | Phyllanthaceae | S |
| 3 | *Actinodaphne bourdillonii* | Lauraceae | T |
| 4 | *Actinodaphne bourneae* | Lauraceae | T |
| 5 | *Actinodaphne malabarica* | Lauraceae | T |
| 6 | *Aglaia barberi* | Meliaceae | T |
| 7 | *Aglaia elaeagnoidea* | Meliaceae | T |
| 8 | *Aglaia indica* | Meliaceae | T |
| 9 | *Aglaia jainii* | Meliaceae | T |
| 10 | *Aglaia lawii* | Meliaceae | T |
| 11 | *Aglaia malabarica* | Meliaceae | T |
| 12 | *Aglaia simplicifolia* | Meliaceae | T |
| 13 | *Agrostistachys borneensis* | Euphorbiaceae | S |
| 14 | *Alseodaphne semecarpifolia* | Lauraceae | H |
| 15 | *Alysicarpus vaginalis* | Fabaceae | H |
| 16 | *Amomum muricatum* | Zingiberaceae | S |
| 17 | *Ancistrocladus heyneanus* | Ancistrocladaceae | S |
| 18 | *Antidesma bunius* | Euphorbiaceae | T |
| 19 | *Archidendron bigeminum* | Fabaceae | T |
| 20 | *Ardisia rhomboidea* | Myrsinaceae | S |
| 21 | *Arenga wightii* | Arecaceae | H |
| 22 | *Argyreia cuneata* | Convolvulaceae | C |
| 23 | *Artocarpus gomezianus* | Moraceae | T |
| 24 | *Artocarpus hirsutus* | Moraceae | T |
| 25 | *Atalantia wightii* | Rutaceae | H |
| 26 | *Atuna travancorica* | Chrysobalanaceae | H |
| 27 | *Baccaurea courtallensis* | Euphorbiaceae | T |
| 28 | *Bambusa arundinacea* | Poaceae | S |
| 29 | *Bauhinia vahlii* | Fabaceae | T |
| 30 | *Beilschmiedia wightii* | Lauraceae | S |
| 31 | *Bombax insigne* | Bombacaceae | C |
| 32 | *Butea monosperma* | Fabaceae | T |
| 33 | *Calophyllum apetalum* | Clusiaceae | T |
| 34 | *Calophyllum trapezifolium* | Clusiaceae | T |
| 35 | *Canthium neilgherrense* | Rubiaceae | S |
| 36 | *Canthium parviflorum* | Rubiaceae | S |
| 37 | *Carissa inermis* | Apocynaceae | H |
| 38 | *Casearia bourdillonii* | Salicaceae | S |
| 39 | *Cayratia pedata* | Vitaceae | C |
| 40 | *Celtis australis* | Cannabaceae | T |
| 41 | *Cinnamomum keralaense* | Lauraceae | T |
| 42 | *Cinnamomum macrocarpum* | Lauraceae | T |
| 43 | *Cinnamomum malabatrum* | Lauraceae | T |
| 44 | *Cinnamomum perrottetii* | Lauraceae | T |
| 45 | *Cinnamomum sulphuratum* | Lauraceae | T |
| 46 | *Cinnamomum tamala* | Lauraceae | T |
| 47 | *Cinnamomum wightii* | Lauraceae | T |
| 48 | *Cleistanthus collinus* | Euphorbiaceae | T |
| 49 | *Colebrookea oppositifolia* | Lamiaceae | S |
| 50 | *Commelina hirsuta* | Commelinaceae | H |
|  |  |  |  |
|  |  |  |  |
|  |  |  |  |
| **Sr. No.** | **Species Name** | **Family** | **Habit** |
| 51 | *Crotalaria calycina* | Fabaceae | H |
| 52 | *Croton malabaricus* | Euphorbiaceae | T |
| 53 | *Cryptocarya bourdillonii* | Lauraceae | T |
| 54 | *Curcuma neilgherrensis* | Zingiberaceae | H |
| 55 | *Cyanotis arcotensis* | Commelinaceae | H |
| 56 | *Cycas circinalis* | Cycadaceae | S |
| 57 | *Dalbergia horrida* | Fabaceae | T |
| 58 | *Dalbergia sissoo* | Fabaceae | C |
| 59 | *Desmodium triquetrum* | Fabaceae | S |
| 60 | *Desmos lawii* | Annonaceae | H |
| 61 | *Dichapetalum gelonioides* | Chailletiaceae | H |
| 62 | *Dimorphocalyx lawianus* | Euphorbiaceae | H |
| 63 | *Dioscorea oppositifolia* | Dioscoreaceae | C |
| 64 | *Diospyros barberi* | Ebenaceae | T |
| 65 | *Diospyros bourdillonii* | Ebenaceae | T |
| 66 | *Diospyros montana* | Ebenaceae | T |
| 67 | *Diospyros nilagirica* | Ebenaceae | T |
| 68 | *Diospyros paniculata* | Ebenaceae | T |
| 69 | *Diospyros pruriens* | Ebenaceae | T |
| 70 | *Diospyros saldanhae* | Ebenaceae | T |
| 71 | *Diplazium esculentum* | Athyriaceae | H |
| 72 | *Dipterocarpus bourdillonii* | Dipterocarpaceae | T |
| 73 | *Dipterocarpus indicus* | Dipterocarpaceae | T |
| 74 | *Discospermum sphaerocarpum* | Rubiaceae | H |
| 75 | *Dolichandrone atrovirens* | Bignoniaceae | T |
| 76 | *Drypetes confertiflorus* | Euphorbiaceae | T |
| 77 | *Drypetes malabarica* | Euphorbiaceae | T |
| 78 | *Drypetes oblongifolia* | Euphorbiaceae | T |
| 79 | *Drypetes venusta* | Euphorbiaceae | T |
| 80 | *Drypetes wightii* | Euphorbiaceae | T |
| 81 | *Dysoxylum malabaricum* | Meliaceae | T |
| 82 | *Elaeocarpus glandulosus* | Elaeocarpaceae | T |
| 83 | *Elaeocarpus munronii* | Elaeocarpaceae | T |
| 84 | *Elaeocarpus recurvatus* | Elaeocarpaceae | T |
| 85 | *Elaeocarpus tuberculatus* | Elaeocarpaceae | T |
| 86 | *Elatostema lineolatum* | Urticaceae | H |
| 87 | *Emilia sonchifolia* | Asteraceae | T |
| 88 | *Entada pursaetha* | Fabaceae | H |
| 89 | *Epiprinus mallotiformis* | Euphorbiaceae | H |
| 90 | *Eragrostis tenella* | Poaceae | H |
| 91 | *Euonymus indicus* | Celastraceae | T |
| 92 | *Evodia lunu-ankenda* | Rutaceae | T |
| 93 | *Ficus beddomei* | Moraceae | T |
| 94 | *Flacourtia indica* | Flacourtiaceae | S |
| 95 | *Garcinia gummi-gutta* | Clusiaceae | S |
| 96 | *Garcinia indica* | Clusiaceae | T |
| 97 | *Garcinia morella* | Clusiaceae | T |
| 98 | *Garcinia spicata* | Clusiaceae | T |
| 99 | *Garcinia talbotii* | Clusiaceae | T |
| 100 | *Gardenia latifolia* | Rubiaceae | T |
|  |  |  |  |
|  |  |  |  |
|  |  |  |  |
|  |  |  |  |
| **Sr. No.** | **Species Name** | **Family** | **Habit** |
| 101 | *Glochidion malabaricum* | Euphorbiaceae | S |
| 102 | *Glochidion neilgherrense* | Euphorbiaceae | T |
| 103 | *Glochidion tomentosum* | Euphorbiaceae | T |
| 104 | *Glossocardia bosvallea* | Asteraceae | S |
| 105 | *Gluta travancorica* | Anacardiaceae | H |
| 106 | *Glycosmis macrocarpa* | Rutaceae | T |
| 107 | *Glycosmis mauritiana* | Rutaceae | S |
| 108 | *Goniothalamus cardiopetalus* | Annonaceae | T |
| 109 | *Gordonia obtusa* | Theaceae | S |
| 110 | *Grewia tiliaefolia* | Tiliaceae | S |
| 111 | *Hardwickia binata* | Fabaceae | T |
| 112 | *Heritiera papilio* | Sterculiaceae | H |
| 113 | *Holigarna arnottiana* | Anacardiaceae | T |
| 114 | *Holigarna beddomei* | Anacardiaceae | T |
| 115 | *Holigarna grahamil* | Anacardiaceae | T |
| 116 | *Holigarna nigra* | Anacardiaceae | S |
| 117 | *Hopea canarensis* | Dipterocarpaceae | T |
| 118 | *Hopea parviflora* | Dipterocarpaceae | T |
| 119 | *Hopea ponga* | Dipterocarpaceae | T |
| 120 | *Hopea racophloea* | Dipterocarpaceae | T |
| 121 | *Hydnocarpus pentandra* | Achariaceae | T |
| 122 | *Ixora brachiata* | Rubiaceae | T |
| 123 | *Ixora elongata* | Rubiaceae | S |
| 124 | *Jasminum malabaricum* | Oleaceae | S |
| 125 | *Kingiodendron pinnatum* | Caesalpiniaceae | S |
| 126 | *Knema attenuata* | Myristicaceae | T |
| 127 | *Lagerstroemia microcarpa* | Lythraceae | T |
| 128 | *Lasianthus acuminatus* | Rubiaceae | S |
| 129 | *Ligustrum gamblei* | Oleaceae | S |
| 130 | *Ligustrum perrottetii* | Oleaceae | T |
| 131 | *Litsea bourdillonii* | Lauraceae | T |
| 132 | *Litsea coriacea* | Lauraceae | T |
| 133 | *Litsea floribunda* | Lauraceae | T |
| 134 | *Litsea glabrata* | Lauraceae | T |
| 135 | *Litsea laevigata* | Lauraceae | S |
| 136 | *Litsea ligustrina* | Lauraceae | S |
| 137 | *Litsea mysorensis* | Lauraceae | T |
| 138 | *Litsea stocksii* | Lauraceae | T |
| 139 | *Litsea wightiana* | Lauraceae | S |
| 140 | *Macaranga indica* | Euphorbiaceae | T |
| 141 | *Mahonia leschenaultii* | Berberidaceae | S |
| 142 | *Mallotus atrovirens* | Euphorbiaceae | T |
| 143 | *Mallotus aureopunctatus* | Euphorbiaceae | T |
| 144 | *Mallotus stenanthus* | Euphorbiaceae | T |
| 145 | *Mallotus tetracoccus* | Euphorbiaceae | T |
| 146 | *Mastixia arborea* | Cornaceae | H |
| 147 | *Maytenus heyneana* | Celastraceae | S |
| 148 | *Meiogyne pannosa* | Annonaceae | S |
| 149 | *Meiogyne ramarowii* | Annonaceae | H |
| 150 | *Memecylon heyneanum* | Memecylaceae | S |
|  |  |  |  |
|  |  |  |  |
|  |  |  |  |
|  |  |  |  |
| **Sr. No.** | **Species Name** | **Family** | **Habit** |
| 151 | *Memecylon malabaricum* | Memecylaceae | T |
| 152 | *Memecylon talbotianum* | Memecylaceae | T |
| 153 | *Mesua ferrea* | Clusiaceae | T |
| 154 | *Myristica fatua* | Myristicaceae | T |
| 155 | *Myristica malabarica* | Myristicaceae | S |
| 156 | *Neolitsea scrobiculata* | Lauraceae | T |
| 157 | *Nothapodytes nimmoniana* | Icacinaceae | T |
| 158 | *Nothopegia beddomei* | Anacardiaceae | T |
| 159 | *Nothopegia heyneana* | Anacardiaceae | T |
| 160 | *Nothopegia racemosa* | Anacardiaceae | T |
| 161 | *Nothopegia travancorica* | Anacardiaceae | H |
| 162 | *Ochna obtusata* | Ochnaceae | S |
| 163 | *Ophiorrhiza brunonis* | Rubiaceae | S |
| 164 | *Ormosia travancorica* | Fabaceae | H |
| 165 | *Orophea uniflora* | Annonaceae | T |
| 166 | *Otonephelium stipulaceum* | Sapindaceae | H |
| 167 | *Palaquium bourdillonii* | Sapotaceae | T |
| 168 | *Palaquium ellipticum* | Sapotaceae | C |
| 169 | *Peristrophe andamanica* | Acanthaceae | S |
| 170 | *Pinanga dicksonii* | Arecaceae | S |
| 171 | *Pittosporum dasycaulon* | Pittosporaceae | T |
| 172 | *Pittosporum neelgherrense* | Pittosporaceae | S |
| 173 | *Poeciloneuron indicum* | Clusiaceae | H |
| 174 | *Pogostemon benghalense* | Lamiaceae | S |
| 175 | *Polyalthia fragrans* | Annonaceae | T |
| 176 | *Psychotria anamallayana* | Rubiaceae | S |
| 177 | *Psychotria flavida* | Rubiaceae | S |
| 178 | *Psychotria nigra* | Rubiaceae | S |
| 179 | *Psychotria octosulcata* | Rubiaceae | C |
| 180 | *Psychotria truncata* | Rubiaceae | H |
| 181 | *Pterospermum reticulatum* | Sterculiaceae | T |
| 182 | *Reinwardtiodendron anamalaiense* | Meliaceae | T |
| 183 | *Rhododendron nilagiricum* | Ericaceae | S |
| 184 | *Sageraea laurifolia* | Annonaceae | T |
| 185 | *Saprosma corymbosum* | Rubiaceae | T |
| 186 | *Saprosma fragrans* | Rubiaceae | T |
| 187 | *Saprosma glomeratum* | Rubiaceae | S |
| 188 | *Schefflera racemosa* | Araliaceae | T |
| 189 | *Shorea robusta* | Dipterocarpaceae | T |
| 190 | *Shorea roxburghii* | Dipterocarpaceae | H |
| 191 | *Spondias pinnata* | Anacardiaceae | H |
| 192 | *Sterculia villosa* | Sterculiaceae | T |
| 193 | *Strychnos dalzellii* | Loganiaceae | S |
| 194 | *Symplocos cochinchinensis* | Symplocaceae | T |
| 195 | *Symplocos foliosa* | Symplocaceae | S |
| 196 | *Symplocos laurina* | Symplocaceae | S |
| 197 | *Symplocos macrophylla* | Symplocaceae | T |
| 198 | *Symplocos monantha* | Symplocaceae | T |
| 199 | *Symplocos pulchra* | Symplocaceae | S |
| 200 | *Syzygium balsameum* | Myrtaceae | T |
|  |  |  |  |
|  |  |  |  |
|  |  |  |  |
|  |  |  |  |
| **Sr. No.** | **Species Name** | **Family** | **Habit** |
| 201 | *Syzygium densiflorum* | Myrtaceae | T |
| 202 | *Syzygium gardneri* | Myrtaceae | T |
| 203 | *Syzygium laetum* | Myrtaceae | S |
| 204 | *Syzygium manii* | Myrtaceae | T |
| 205 | *Syzygium mundagam* | Myrtaceae | T |
| 206 | *Tabernaemontana gamblei* | Apocynaceae | T |
| 207 | *Tabernaemontana heyneana* | Apocynaceae | H |
| 208 | *Tricalysia apiocarpa* | Rubiaceae | T |
| 209 | *Turpinia malabarica* | Sapindaceae | T |
| 210 | *Vateria indica* | Dipterocarpaceae | T |
| 211 | *Vateria macrocarpa* | Dipterocarpaceae | T |
| 212 | *Ventilago maderaspatana* | Rhamnaceae | T |
| 213 | *Vepris bilocularis* | Rutaceae | H |
| 214 | *Vernonia arborea* | Asteraceae | H |
| 215 | *Vernonia gossypina* | Asteraceae | H |
| 216 | *Viola pilosa* | Violaceae | H |
| 217 | *Zingiber cernuum* | Zingiberaceae | S |

|  | | | |
| --- | --- | --- | --- |
| **Endemic plant species of Indo Burma** | | | |
| **Sr. No.** | **Species Name** | **Family** | **Habit** |
| 1 | *Achyranthes aspera* | Amaranthaceae | H |
| 2 | *Alstonia kurzii* | Apocynaceae | T |
| 3 | *Alysicarpus vaginalis* | Fabaceae | H |
| 4 | *Amomum aculeatum* | Zingiberaceae | H |
| 5 | *Antidesma bunius* | Euphorbiaceae | T |
| 6 | *Aquilaria malaccensis* | Thymelaeceae | H |
| 7 | *Ardisia andamanica* | Myrsinaceae | S |
| 8 | *Argyreia roxburghii* | Convolvulaceae | H |
| 9 | *Artocarpus chama* | Moraceae | S |
| 10 | *Arundinaria falcata* | Poaceae | S |
| 11 | *Bambusa tulda* | Poaceae | T |
| 12 | *Bauhinia vahlii* | Fabaceae | T |
| 13 | *Bergenia ciliata* | Saxifragaceae | H |
| 14 | *Blachia andamanica* | Euphorbiaceae | T |
| 15 | *Boehmeria rugulosa* | Urticaceae | S |
| 16 | *Bombax insigne* | Bombacaceae | C |
| 17 | *Brassaiopsis mitis* | Araliaceae | T |
| 18 | *Buchanania splendens* | Anacardiaceae | H |
| 19 | *Butea monosperma* | Fabaceae | T |
| 20 | *Calamus andamanicus* | Arecaceae | S |
| 21 | *Calamus palustris* | Arecaceae | T |
| 22 | *Calamus pseudorivalis* | Arecaceae | S |
| 23 | *Calamus viminalis* | Arecaceae | S |
| 24 | *Casearia andamanica* | Salicaceae | S |
| 25 | *Cedrus deodara* | Pinaceae | T |
| 26 | *Cinnamomum tamala* | Lauraceae | T |
| 27 | *Cinnamomum wightii* | Lauraceae | T |
| 28 | *Coffea benghalensis* | Rubiaceae | S |
| 29 | *Cryptocarya amygdalina* | Lauraceae | T |
| 30 | *Dalbergia sissoo* | Fabaceae | C |
| 31 | *Daphne papyracea* | Thymelaeaceae | T |
| 32 | *Dendrocalamus hamiltonii* | Poaceae | S |
| 33 | *Desmos lawii* | Annonaceae | H |
| 34 | *Dinochloa andamanica* | Poaceae | H |
| 35 | *Dioscorea belophylla* | Dioscoreaceae | C |
| 36 | *Dioscorea bulbifera* | Dioscoreaceae | C |
| 37 | *Diospyros montana* | Ebenaceae | T |
| 38 | *Diplazium esculentum* | Athyriaceae | H |
| 39 | *Dipterocarpus grandiflorus* | Dipterocarpaceae | T |
| 40 | *Elaeocarpus glandulosus* | Elaeocarpaceae | T |
| 41 | *Elatostema lineolatum* | Urticaceae | H |
| 42 | *Entada pursaetha* | Fabaceae | H |
| 43 | *Evodia lunu-ankenda* | Rutaceae | T |
| 44 | *Ganophyllum falcatum* | Sapindaceae | T |
| 45 | *Gaultheria fragrantissima* | Ericaceae | T |
| 46 | *Globba multiflora* | Zingiberaceae | H |
| 47 | *Globba racemosa* | Zingiberaceae | T |
| 48 | *Glochidion andamanicum* | Euphorbiaceae | T |
| 49 | *Glochidion calocarpum* | Euphorbiaceae | T |
| 50 | *Hedychium spicatum* | Zingiberaceae | H |
|  |  |  |  |
|  |  |  |  |
|  |  |  |  |
| **Sr. No.** | **Species Name** | **Family** | **Habit** |
| 51 | *Hedychium venustum* | Zingiberaceae | H |
| 52 | *Holigarna grahamii* | Anacardiaceae | T |
| 53 | *Impatiens chinensis* | Balsaminaceae | H |
| 54 | *Ixora barbata* | Rubiaceae | T |
| 55 | *Ixora brunnescens* | Rubiaceae | S |
| 56 | *Ixora grandifolia* | Rubiaceae | S |
| 57 | *Lagerstroemia hypoleuca* | Lythraceae | T |
| 58 | *Lasianthus tubiferus* | Rubiaceae | S |
| 59 | *Litsea kurzii* | Lauraceae | T |
| 60 | *Macaranga indica* | Euphorbiaceae | T |
| 61 | *Maesa andamanica* | Myrsinaceae | S |
| 62 | *Mallotus tetracoccus* | Euphorbiaceae | T |
| 63 | *Mangifera andamanica* | Anacardiaceae | T |
| 64 | *Mesua ferrea* | Clusiaceae | T |
| 65 | *Musa velutina* | Musaceae | S |
| 66 | *Myristica andamanica* | Myristicaceae | T |
| 67 | *Ophiorrhiza wallichii* | Rubiaceae | H |
| 68 | *Persea gamblei* | Lauraceae | T |
| 69 | *Phlogacanthus tubiflorus* | Acanthaceae | T |
| 70 | *Pinus roxburghii* | Pinaceae | T |
| 71 | *Polyalthia fragrans* | Annonaceae | T |
| 72 | *Polyalthia parkinsonii* | Annonaceae | T |
| 73 | *Premna bengalensis* | Verbenaceae | T |
| 74 | *Premna milleflora* | Verbenaceae | T |
| 75 | *Pseuduvaria prainii* | Annonaceae | T |
| 76 | *Psychotria adenophylla* | Rubiaceae | H |
| 77 | *Psychotria platyneura* | Rubiaceae | C |
| 78 | *Pyrus pashia* | Rosaceae | T |
| 79 | *Rhododendron arboreum* | Ericaceae | S |
| 80 | *Rhododendron barbatum* | Ericaceae | S |
| 81 | *Sapium eugeniaefolium* | Euphorbiaceae | S |
| 82 | *Sarcosperma arboreum* | Sapotaceae | C |
| 83 | *Saurauia roxburghii* | Actinidiaceae | S |
| 84 | *Schima wallichii* | Theaceae | S |
| 85 | *Shorea assamica* | Dipterocarpaceae | T |
| 86 | *Shorea robusta* | Dipterocarpaceae | T |
| 87 | *Sloanea sterculiacea* | Elaeocarpaceae | H |
| 88 | *Spatholobus parviflorus* | Fabaceae | H |
| 89 | *Spondias pinnata* | Anacardiaceae | H |
| 90 | *Sterculia villosa* | Sterculiaceae | T |
| 91 | *Strobilanthes andamanensis* | Acanthaceae | H |
| 92 | *Symplocos cochinchinensis* | Symplocaceae | T |
| 93 | *Syzygium kurzii* | Myrtaceae | T |
| 94 | *Tabernaemontana heyneana* | Apocynaceae | H |
| 95 | *Tetrastigma planicaule* | Vitaceae | C |
| 96 | *Xanthophyllum andamanicum* | Polygalaceae | S |
