# Supplementary material for: Future of Endemic Flora of Biodiversity Hotspots in India
Source: PLoS One. 2014 Dec 12;9(12):e115264. doi: 10.1371/journal.pone.0115264 (PMC4264876; doi:10.1371/journal.pone.0115264)
Supplement: S2 Table — List of predictor variables. (DOC) [file pone.0115264.s002.doc]

**Supplementary Information**

| **Sr. No.** | **Climate variable** |
| --- | --- |
| 1 | Annual mean temperature (ºC.) |
| 2 | Temperature seasonality |
| 3 | Temperature annual range (ºC.) |
| 4 | Mean temperature of warmest quarter (ºC.) |
| 5 | Annual precipitation (mm) |
| 6 | Precipitation seasonality |
| 7 | Annual mean radiation (Wm-2) |
| 8 | Annual mean moisture index |
| 9 | Mean moisture index of wettest quarter |
| 10 | Mean moisture index of driest quarter |
| **Sr. No.** | **Disturbance variable** |
| 1 | Human population density |
| 2 | Forest fragmentation |
| **Sr. No.** | **Physiography variable** |
| 1 | Terrain complexity |

Table S2 List of predictor variables
