# Supplementary material for: Future of Endemic Flora of Biodiversity Hotspots in India
Source: PLoS One. 2014 Dec 12;9(12):e115264. doi: 10.1371/journal.pone.0115264 (PMC4264876; doi:10.1371/journal.pone.0115264)
Supplement: S1 File — Forest fragmentation. (DOCX) [file pone.0115264.s003.docx]

**Supplementary Information S1**

**Forest fragmentation** (adapted from Roy et al., 2013)

The number of patches of forest and non-forest types per unit area derived based on the vegetation type map was taken as a working definition (Roy et al., 2012). A user grid cell of size *n* × *n* (where *n* represents the length of the grid along one side, say 500 m) was convolved with the spatial data layer of forest and non-forest grids. The number of forest patches within the grid cells was derived. The model was designed as a software module in the SPLAM software package. The process was repeated by moving the grid cell through the entire layer. An output layer with patch numbers was derived, and an associated lookup table was generated that rescales the normalized data of the patches per cell in the range 0–10. The fragmentation was computed using the equation,

Frag = *f* (*n*F, *n*NF),

where, Frag is the fragmentation; *n* the number of patches, *F* the forest patches and NF the non-forest patches. Fragmentation index ranges from 1 to 7. The cells of this map were further classified as intact (index value 1, which means that there is only one forest pixel in the 500 m × 500 m window), low (index value 2, which means there are two forest patches in the window), medium (index value 3) and high (index values 4–7). The classification of the fragmentation map cells as intact, low, medium and high is based on histogram clustering and expert knowledge.
